# Vitamin A and Feeding Statuses Modulate the Insulin-Regulated Gene Expression in Zucker Lean and Fatty Primary Rat Hepatocytes

**DOI:** 10.1371/journal.pone.0100868

**Published:** 2014-08-08

**Authors:** Wei Chen, Meredith L. Howell, Yang Li, Rui Li, Guoxun Chen

**Affiliations:** Department of Nutrition, University of Tennessee at Knoxville, Knoxville, Tennessee, United States of America; Boston University School of Medicine, United States of America

## Abstract

Unattended hepatic insulin resistance predisposes individuals to dyslipidemia, type 2 diabetes and many other metabolic complications. The mechanism of hepatic insulin resistance at the gene expression level remains unrevealed. To examine the effects of vitamin A (VA), total energy intake and feeding conditions on the insulin-regulated gene expression in primary hepatocytes of Zucker lean (ZL) and fatty (ZF) rats, we analyze the expression levels of hepatic model genes in response to the treatments of insulin and retinoic acid (RA). We report that the insulin- and RA-regulated glucokinase, sterol regulatory element-binding protein-1c and cytosolic form of phosphoenolpyruvate carboxykinase expressions are impaired in hepatocytes of ZF rats fed chow or a VA sufficient (VAS) diet ad libitum. The impairments are partially corrected when ZF rats are fed a VA deficient (VAD) diet ad libitum or pair-fed a VAS diet to the intake of their VAD counterparts in non-fasting conditions. Interestingly in the pair-fed ZL and ZF rats, transient overeating on the last day of pair-feeding regimen changes the expression levels of some VA catabolic genes, and impairs the insulin- and RA-regulated gene expression in hepatocytes. These results demonstrate that VA and feeding statuses modulate the hepatic insulin sensitivity at the gene expression level.

## Introduction

The liver is critical for glucose and lipid homeostasis, which is achieved partially through the regulation of hepatic gene expression in response to hormonal and nutritional stimuli [1], [2]. The disturbance of these responses may lead to the development of metabolic diseases, including obesity and type 2 diabetes [1]. Even though the pathogenesis of these diseases has not been fully elucidated, insulin resistance is a common feature [3]. Current theory postulates that overnutrition triggers system inflammation, dysregulates lipid metabolism, and alters gastrointestinal microbiota, all of which may interplay and lead to impaired insulin action in the body [4].

The insulin-regulated hepatic metabolism is partially attributed to the regulation of hepatic genes expression. For instance, in primary rat hepatocytes, insulin induces the glucokinase (GCK, gene *Gck*) expression to promote glycolysis and suppresses the phosphoenolpyruvate carboxykinase (PEPCK-C, gene *Pck1*) expression to reduce gluconeogenesis [5], [6]. Insulin also induces hepatic lipogenesis via inducing sterol regulatory element-binding protein 1c (SREBP-1c, gene *Srebp-1c*) expression. SREBP-1c stimulates the expression of hepatic lipogenic genes, such as acetyl-coenzyme A carboxylase and fatty acid synthase [7]. In the insulin-resistant liver, insulin is unable to suppress the expression of gluconeogenic genes. But it is able to concurrently induce the expression of lipogenic genes [2]. This phenomenon demonstrates the coexistence of hyperglycemia and hyperlipidemia in type 2 diabetes.

As an essential and lipophilic micronutrient, vitamin A (VA, retinol) is implicated in the regulation of glucose and lipid metabolism [1]. Two stepwise cytosolic enzymatic reactions convert retinol to retinal and then into retinoic acid (RA, all-*trans* RA or 9-*cis* RA), which then enters the nucleus and modules gene expression through the activation of retinoid acid receptors and retinoid X receptors [8]. We have reported that the insulin-regulated gene expression is impaired in primary hepatocytes from Zucker fatty (ZF) rats fed ad libitum [9]. Recent reports by our group also show that RA synergizes with insulin to induce the *Gck* and *Srebp-1c* expressions in primary rat hepatocytes [10], [11]. RA attenuates the insulin-suppressed *Pck1* expression in the same cells [12]. These data suggest that micronutrient status can affect hepatic insulin sensitivity at the gene expression level. However, the roles of caloric intake and micronutrient composition in the hepatic insulin resistance at the gene expression level have not been investigated.

Here, we chose Zucker lean (ZL) and ZF rats as insulin sensitive and resistant models, respectively, to investigate the effects of energy intake and VA status on the hepatic insulin sensitivity at the gene expression level. We demonstrate that VA deficiency partially corrects the impaired insulin-regulated gene expression in ZF primary hepatocytes. More importantly, transient overeating in ZL and ZF rats causes the impairment of insulin-regulated gene expression in their primary hepatocytes.

## Materials and Methods

### Reagents

The reagents for primary hepatocyte isolation and culture including Medium 199, Dulbecco's Modification of Eagle Medium (DMEM), liver perfusion buffer and liver digest buffer were obtained from Invitrogen (Carlsbad, CA). For RNA extraction, RNA STAT-60 was purchased from TEL-TEST (Friendswood, TX). The reagents for cDNA synthesis and real-time PCR were obtained from Applied Biosystems (Foster city, CA). All real-time PCR primer sets used in this study were synthesized by Sigma-Aldrich (St. Louis, MO). All other reagents and materials were purchased from Fisher Scientific (Pittsburgh, PA) unless described otherwise.

### Animals and rodent diets

Zucker rats (Harlan Laboratories) were bred and housed on a 12 hour light-dark cycle under constant temperature and humidity in the animal facility at University of Tennessee at Knoxville. Breeding pairs were kept on Teklad rodent chow (#8640, Harlan Laboratories, Indianapolis, IN). Weaned male ZL (fa/+ or +/+) and ZF (fa/fa) rats (21 days old) were fed experimental diets for 8 weeks before primary hepatocyte isolation. Synthetic VA sufficient (VAS, #5755, 22.1 IU/g VA) and VA deficient (VAD, #5822, 0 IU/g VA) diets were isocaloric diets from TestDiet (Richmond, VA). All procedures (Protocol #1256, #1863) were approved by the Institutional Animal Care and Use Committee at the University of Tennessee at Knoxville.

### Pair-feeding study

Male ZL or ZF rats (VAD-AD group, and VAD ad libitum, 5 rats per group) had free access to the VAD diet and water for 56 days. The body mass and food intake were recorded every 2 days. Primary hepatocytes were isolated on Day 56. Two pair-feeding groups were set up to circumvent a potential caveat on the last day of pair-feeding. Since VAD animals consume significantly less diet than their VAS counterparts in ad libitum feeding condition [13]–[15], the VAS pair-fed rats would conceivably consume the last day's ration rapidly. This caveat would result in a fasting status in these rats and affect the analysis of insulin-regulated hepatic gene expression. Therefore, 10 rats with matching genotypes were paired up with rats in VAD-AD group according to weaning body mass. They were pair-fed the VAS diet for 55 days to the intakes of their VAD-AD counterparts. During the course of pair-feeding, unfinished food pellets were rolled over to ensure that equal total calories were provided. The body mass and food intake of these rats were recorded daily. On Day 56, one group, VAS-PF-AD (VAS pair-feeding last day ad libitum), was allowed to have free access to the VAS diet to prevent fasting energy status. The other group, VAS-PF-4M (VAS pair-feeding last day 4 meals), was fed the VAS diet to the amount consumed by the VAD-AD group. One fourth of the ration was provided to VAS-PF-4M rats every 6 hours, so that they remained non-fasted energy status till the end of the study. Before primary hepatocyte isolation, the tail tip whole blood glucose was measured using a LifeScan OneTouch Ultramini glucometer (Milpitas, CA).

### Primary hepatocyte isolation

The primary hepatocytes were isolated according to previously described protocol [9]. The rat was euthanized by primary carbon dioxide asphyxiation, and then secondary cervical dislocation according to the protocol. A peristaltic pump with the flow rate of 10 ml/min was set up to infuse about 120 ml liver perfusion buffer and 120 ml liver digestion buffer. A catheter connected to the pump was inserted into the portal vein. The inferior vena cava was punctured to allow the outflow of blood and buffers. The liver was then excised and put into a cell culture dish containing liver digestion buffer to remove connective tissues. The released hepatocytes were filtered through a 100 µm cell strainer and collected by 50× g centrifugation for 3 minutes. The hepatocytes were washed twice with high glucose DMEM containing 8% fetal bovine serum, 1% penicillin/streptomycin. Isolated hepatocytes were seeded on 60-mm collagen type I coated dishes at 2×10^6^ cells per dish and incubated in high glucose DMEM containing 8% fetal bovine serum, 1% penicillin/streptomycin at 37°C and 5% CO_2_ for 3 hours. The attached hepatocytes were washed once with PBS and pretreated in medium A (Medium 199 with 100 nM dexamethasone, 100 nM 3,3′,5-triiodo-L-thyronine (T3), and 1% penicillin/streptomycin) containing 1 nM insulin at 37°C and 5% CO2 for 14–16 hours.

### Insulin and RA treatments, RNA extraction and real-time PCR

The pretreated hepatocytes were washed once with PBS, and then treated for 6 hours at 37°C and 5% CO2 with medium A containing indicated concentrations of insulin (0 nM to 100 nM) with or without 5 µM RA. The methods for total RNA extraction and cDNA synthesis were described elsewhere [16]. In essence, total RNA was extraction from the treated hepatocytes with RNA STAT-60 according to the manufacture's protocol. The contaminated DNA in RNA samples was removed using the DNA-free kit. First strand cDNA was synthesized with 2 µg total RNA by cDNA synthesis kit. The gene expression level was determined by real-time PCR with respective primer sets, and normalized to the mRNA level of ribosomal gene 36B4. The data were presented as either minus Δ cycle threshold (Ct) or the induction fold (ΔΔCt) for which the control treatment group was arbitrarily set as 1.

### Statistical analysis

Statistical analyses were performed using SPSS 19.0 software. Student t-test was used to compare the means between two treatments. One-way ANOVA with LSD post-hoc test was used to compare the means of three or more treatments. Two-way ANOVA with Bonferroni's post-hoc test was used to determine the effects of diets and genotypes on hepatic gene expression. Data were presented as means ± S.E.M. The number of experiments indicates hepatocyte isolations from different animals. A p value less than 0.05 is considered statistically significant.

## Results

### The insulin- and RA-regulated *Gck*, *Pck1*, and *Srebp-1c* expressions were impaired in primary hepatocytes from ZF rats fed chow ad libitum

**Figure 1:**
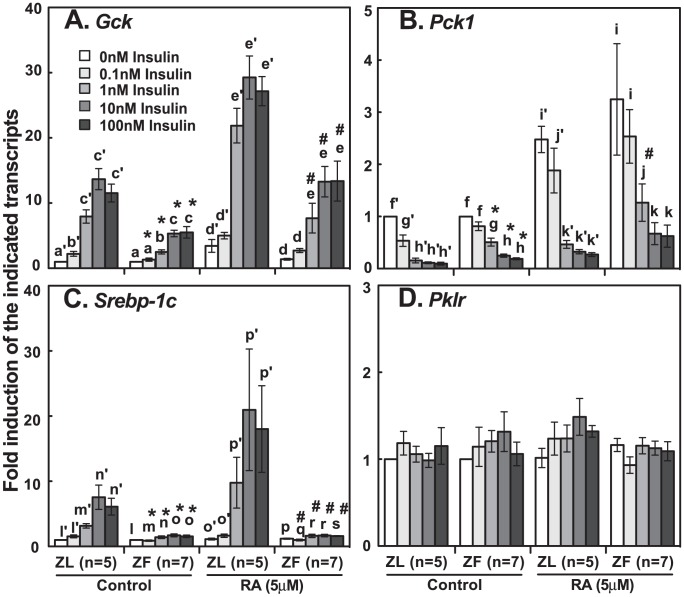
Impaired insulin-regulated gene expression in primary hepatocytes of ZF rats fed chow ad libitum. ZL and ZF rats were fed standard chow for eight weeks before primary hepatocytes were harvested. The primary hepatocytes were incubated in medium A with increasing concentrations of insulin (0 nM to 100 nM) in the absence or presence of RA (5 µM) for 6 hours. Total RNA was extracted, synthesized into cDNA, and then subjected to real-time PCR analysis for the expression levels of *Gck* (A), *Pck1* (B), *Srebp-1c* (C), and *Pklr* (D). The expression level of each gene transcript in ZL or ZF hepatocytes treated with vehicle control was arbitrarily set to 1. The data were expressed as fold induction. The numbers of hepatocyte isolation are presented in parenthesis. All p<0.05; for (A), a′<b′<c′, a<b<c, d′<e′, d<e; for (B), f′>g′>h′, f>g>h, i′>j′, i>k; for (C), l′<m′<n′, m<o, p′<q′, r<s using one-way ANOVA; * or # for comparing ZL or ZF at corresponding treatments using Student's t-test, respectively.

We compared the insulin-regulated gene expression in primary hepatocytes from ZL and ZF rats fed chow ad libitum. Insulin dose-dependently induced the *Gck* and *Srebp-1c* expressions (Figure 1A and 1C), and suppressed the *Pck1* expression (Figure 1B) in ZL hepatocytes. RA (5 µM) synergized with insulin to induce the *Gck* and *Srebp-1c* expressions (Figure 1A and 1C). The elevated *Pck1* level in the presence of RA was still lowered by insulin at 1 nM or higher (Figure 1B). Comparably in ZF hepatocytes, the fold inductions of *Gck* expression by insulin (marked by *) and RA + insulin (marked by #) at the corresponding concentrations were significantly lower than that in ZL hepatocytes (Figure 1A). The inductions of *Srebp-1c* by insulin (0.1 to 100 nM) and RA + insulin (0.1 to 100 nM) were abolished in ZF hepatocytes (Figure 1C). Additionally, the insulin-mediated suppression of *Pck1* was less profound in ZF hepatocytes compared with that in ZL hepatocytes (Figure 1B, marked by * and #, and Figure S1 in File S1). Furthermore, the expressions of liver type pyruvate kinase gene (*Pklr*) in primary hepatocytes from either ZL or ZF rats were not affected by insulin and RA treatments (Figure 1D). These data confirmed our previous observations [9] and demonstrated the hepatic insulin resistance at the gene expression level in ZF rats fed chow ad libitum.

### The insulin- and RA-regulated *Gck*, *Pck1* and *Srebp-1c* expressions were partially recovered in the primary hepatocytes from ZF rats fed a VAD diet ad libitum

**Figure 2:**
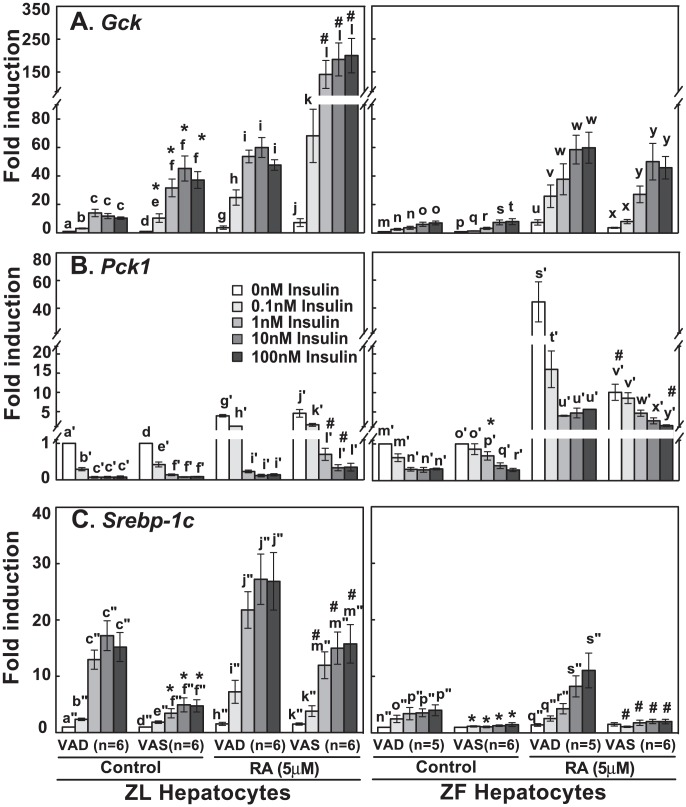
VAD diet partially recovered the impaired insulin-regulated gene expression in ZF primary rat hepatocytes. ZL and ZF rats were fed either a VAS or VAD diet for 8 weeks. Primary hepatocytes were isolated from rats in ad libitum. Cells were incubated in medium A with increasing concentrations of insulin (0 nM to 100 nM) in the absence or presence of RA (5 µM) for 6 hours. Total RNA was extracted, synthesized into cDNA, and then subjected to real-time PCR analysis for the expression levels of *Gck* (A), *Pck1* (B), and *Srebp-1c* (C). The data were expressed as fold induction. The gene transcript levels from the no treatment group (0 nM insulin, no RA) for both ZL and ZF were arbitrarily set to 1. The numbers of hepatocyte isolation are presented in parenthesis. All p<0.05; for (A), a<b<c, d<e<f, g<h<i, j<k<l, m<o, p<r/s/t, q<s/t, r<t, x<y; for (B), a′>b′>c′, d′>e′>f′, g′>h′>i, j′>k′>l′, m′>n′, o′>q′/r′, p′>r′, s′>t′>u′, v′>x′/y′, w′>y′; for (C), a″<b″<c″, d″<e″<f″, h″<i″<j″, k″<m″, n″<p″, q″<s″, using one-way ANOVA. * or # for comparing ZL and ZF at corresponding treatments using Student's t-test, respectively.

VA deficiency reduced food intake, body mass, plasma insulin and triglyceride levels in Zucker rats [9]. Hence, it may attenuate the impairment of the insulin-regulated gene expression in ZF rat hepatocytes. Figure 2A (left panel) showed that, in both VAD and VAS ZL primary hepatocytes, insulin dose-dependently induced the *Gck* expression (first and second column clusters), and RA synergized with insulin to induce its expression (third and fourth column clusters). The fold inductions of *Gck* expression by insulin (marked by *) or by RA + insulin (marked by #) in the VAS ZL hepatocytes were significantly higher than that in VAD ZL hepatocytes at the corresponding insulin concentrations. In comparison, the insulin-induced *Gck* expression was impaired in both VAD and VAS ZF hepatocytes regardless of RA (Figure 2A, right panel).

Insulin dose-dependently suppressed the *Pck1* expression in both VAD and VAS ZL hepatocytes (Figure 2B, left panel, first and second column clusters). RA induced the *Pck1* expression. Insulin at 1 to 100 nM suppressed the *Pck1* expression by over 90% in both VAD and VAS ZL hepatocytes (third and fourth column clusters, Figure S2B in File S1). The net *Pck1* mRNA levels in the presence of RA were higher than that in the absence of RA. On the other hand, the insulin-suppressed *Pck1* expression was impaired in VAS ZF hepatocytes. Insulin at 0.1 nM or 1 nM only suppressed 10% or 40% of the *Pck1* expression level, respectively. However, in VAD ZF hepatocytes, the suppression of *Pck1* expression by 0.1 nM or 1 nM insulin was 40% or 60%, respectively, suggesting improved regulation by insulin (Figure 2B, right panel, first and second column clusters, Figure S2A in File S1). RA induced the *Pck1* expression in VAD and VAS ZF hepatocytes (right panel, third and fourth column clusters). Insulin at 0.1 nM or 1 nM respectively suppressed the *Pck1* expression by 50% or 70% in VAD ZF hepatocytes, which was greater than 20% or 50% in VAS ZF hepatocytes (Figure S2B in File S1).

Insulin dose-dependently induced the *Srebp-1c* expression, and RA synergized with insulin to induce its expression in both VAD and VAS ZL hepatocytes (Figure 2C, left panel). The fold inductions of *Srebp-1c* expression by insulin (marked by *) or by insulin and RA (marked by #) in VAS ZL hepatocytes were significantly lower than that in VAD ZL hepatocytes. In VAS ZF hepatocytes, the insulin-induced *Srebp-1c* expression in the absence or presence of RA was abolished (Figure 2C, right panel). Interestingly in VAD ZF hepatocytes, insulin (1 to 100 nM) partially regained the ability to induce the *Srebp-1c* expression, showing improved regulation (first and third cluster).

### ZL or ZF rats pair-fed the VAS diet tended to overeat when sufficient amount of diet was provided

**Figure 3:**
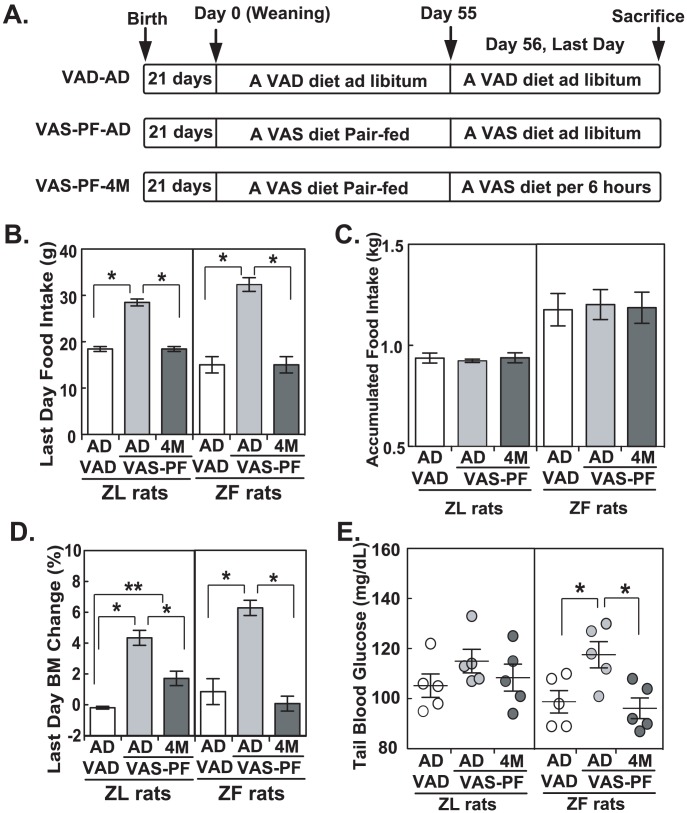
VAS-PF-AD ZL and ZF overate on the last day when sufficient VAS diet was provided. (A) The schematic graph of the setup for the pair-feeding regimen. VAD ad libitum (VAD-AD, n = 5), VAS pair-feeding last day ad libitum (VAS-PF-AD, n = 5), VAS pair-feeding last day 4 meals (VAS-PF-4M, n = 5) (B) Tail tip whole blood glucose from ZL and ZF rats before sacrifice. Each circle represents a value from an individual animal. The bars represent mean ± S.E.M. * Indicates p<0.05 using one-way ANOVA with LSD post-hoc test. (C) The accumulative food intake for ZL and ZF rats over 56 days of pair-feeding regimen. (D) The food intake and (E) body mass change for ZL and ZF rats on day 56 of the pair-feeding regimen. * Indicates p<0.05 using one-way ANOVA with LSD post-hoc test.

Rats fed a VAD diet after weaning reduced food intakes at around 5 weeks [13]. To determine how total and acute food intakes affect the insulin-regulated hepatic gene expression, an 8-week pair-feeding experiment was designed. Rats were divided into three groups, VAD-AD, VAS-PF-AD and VAS-PF-4M (Figure 3A, see Materials and Methods for detail). Figure 3B shows that VAS-PF-AD ZL and ZF rats ingested 54% and 112% more food on the last day than VAD-AD ZL and ZF rats did, respectively, demonstrating the overeating of VAS-PF-AD rats. The overeating of VAS-PF-AD rats did not significantly increase the total caloric intake over the entire pair-feeding period compared to VAD-AD and VAS-PF-4M counterparts (Figure 3C). Correspondingly, VAS-PF-AD ZL and ZF rats gained 4% and 6% of body mass on the last day, respectively (Figure 3D). Before sacrifice, the tail tip whole blood glucose levels of VAS-PF-AD ZF rats were significantly higher than those of VAD-AD and VAS-PF-4M rats (Figure 3E).

### ZL and ZF rats pair-fed the VAS diet had higher body mass and feed efficiency than their VAD fed counterparts after the reduction of food intake

**Figure 4:**
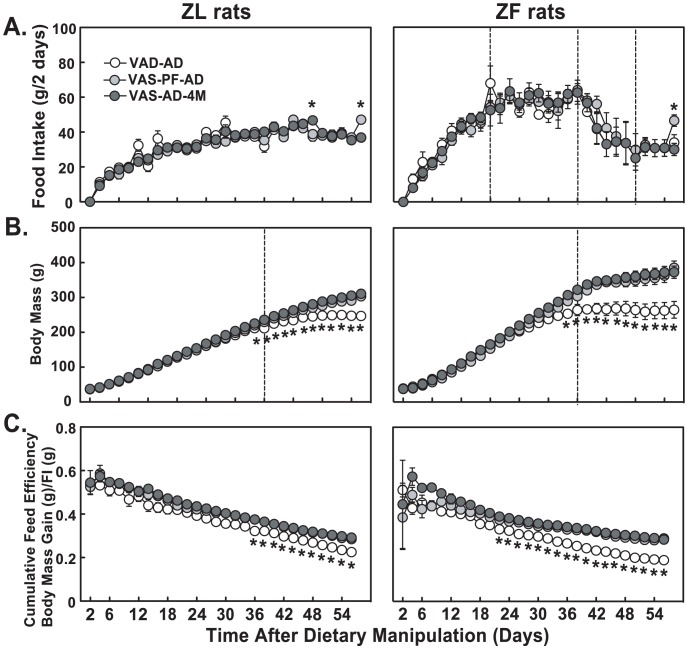
The food intake, body mass and cumulative feed efficiency over 56 days of dietary manipulation. (A) Two-day food intake of ZL and ZF on VAD ad libitum (VAD-AD, n = 5), VAS-Pair-feeding Last Day ad libitum (VAS-PF-AD, n = 5), VAS-Pair-feeding Last Day 4 meals (VAS-PF-4M, n = 5) (B) Body mass of ZL and ZF on VAD AD, VAS-PF AD and VAS-PF 4M. (C) Cumulative feed efficiency (unit weight gain per unit diet consumed) of ZL and ZF on VAD-AD, VAS-PF-AD and VAS-PF-4M. * Indicates p<0.05 using one-way ANOVA with LSD post-hoc test.

During the 56-day pair-feeding regimen, the two-day food intake of VAD-AD ZL rats gradually increased from ∼10 g initially to ∼40 g on days 33–34, and kept relatively stable until days 41–42 (Figure 4A, left panel). As anticipated [13]–[15], the food intake then started to decline until the end of the dietary manipulation. The two-day food intake of VAD-AD ZF rats quickly rose from ∼10 g initially to ∼60 g on days 17–18, leveled off between days 20 to 34, started to decline on days 35–36, reached ∼30 g on days 47–48, and leveled off again until the end of the dietary manipulation (right panel). During the same period, all VAS pair-fed ZL and ZF rats received the same amount of the isocaloric VAS diet to match the energy intake of VAD-AD rats. The body mass of VAD-AD ZL and ZF rats ceased to increase after 36 days of dietary manipulation (Figure 4B). From day 34, the body mass of VAD-AD ZL or ZF rats was significantly lower than that of VAS-PF ZL or ZF rats, respectively (marked by *). Furthermore, the cumulative feed efficiency of all three groups decreased over time. However, VAS-PF ZL or ZF rats had significantly higher feed efficiency than VAD-AD ZL or ZF rats after 36 or 22 days, respectively (Figure 4C).

### Transient overeating impaired the insulin-induced *Gck* expression in primary hepatocytes of VAS-PF-AD ZL and ZF rats

**Figure 5:**
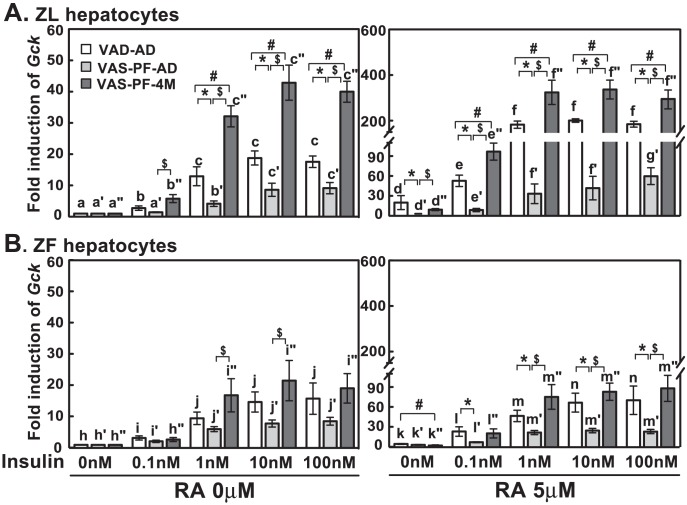
Insulin-induced *Gck* expression was attenuated in hepatocytes from VAS-PF-AD, but not VAS-PF-4M rats. The primary hepatocytes were treated by insulin (0 nM to 100 nM) with or without RA (5 µM) in medium A for 6 hours. The expression level of *Gck* was determined by real-time PCR analysis, and the data were expressed as fold induction. The gene transcript levels from the no treatment group (0 nM insulin, no RA) for both ZL and ZF were arbitrarily set to 1. All p<0.05; for (A), a<b<c, a′<b′<c′, a″<b″<c″, d<e<f, d′<f′/g′, e′<g′, d″<e″<f″; for (B), h<i<j, h′<i′, h″<i″<j″, k<l<m, j′<k′<l′, k″<i″<m″, using one way ANOVA. *, #, and $ for comparing the effects of dietary manipulations at any treatment using one way ANOVA.

In ZL hepatocytes, insulin dose-dependently induced the *Gck* expression (Figure 5A). Without or with RA, the fold inductions by insulin in VAS-PF-AD ZL hepatocytes were significantly lower than that in VAD-AD hepatocytes (marked by *) and VAS-PF-4M ZL hepatocytes (marked by $), suggesting an impaired insulin regulation. Additionally, the fold inductions by insulin in VAD-AD ZL hepatocytes were lower than that of VAS-PF-4M ZL hepatocytes without or with RA (marked by #). These results match the data shown in Figure 2A and indicate that VA status of ZL rats affects the insulin-stimulated *Gck* expression in their hepatocytes.

In ZF hepatocytes, insulin dose-dependently induced the *Gck* expression regardless of RA (Figure 5B). Without RA, the fold inductions by insulin (1 and 10 nM) in VAS-PF-AD ZF hepatocytes were significantly lower than that in VAS-PF-4M ZF hepatocytes (Figure 5B, left panel, marked by $). With RA (Figure 5B, right panel), the fold inductions by insulin in VAS-PF-AD ZF hepatocytes were significantly lower than that in VAD-AD (0.1 to 100 nM, marked by *) and VAS-PF-4M (1 to 100 nM, marked by $) ZF hepatocytes. These data demonstrate the impairment of the insulin-induced *Gck* expression in VAS-PF-AD ZF hepatocytes.

### The insulin-suppressed *Pck1* expression was partially impaired in primary hepatocytes of VAS-PF-AD ZL rats

**Figure 6:**
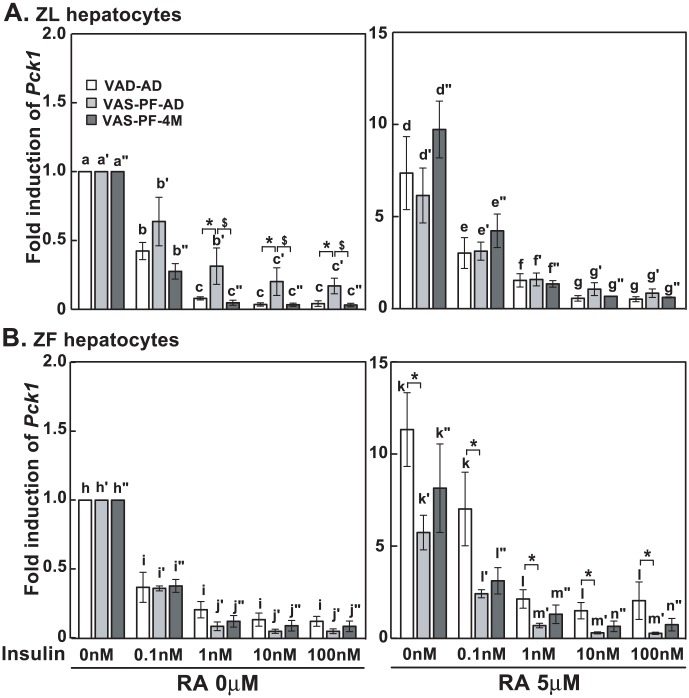
Transient overfeeding of the VAS diet impaired the insulin-suppressed *Pck1* expression in hepatocytes of VAS-PF-AD ZL rats. The primary hepatocytes were treated by insulin (0 nM to 100 nM) with or without RA (5 µM) in medium A for 6 hours. The expression level of *Pck1* was determined by real-time PCR analysis, and the data were expressed as fold induction. The gene transcript levels from the no treatment group (0 nM insulin, no RA) for both ZL and ZF were arbitrarily set to 1. All p<0.05; for (A), a>b>c, a′>c′, a″>b″>c″, d>e>g, d>f, d′>f′/g′, e′>g′, d″>e″>f″>g″; for (B), h>i, h′>i′>j′, h″>i″>j″, k>l, k′>l′>m′, k″>l″, using one way ANOVA. *, #, and $ for comparing the effects of dietary manipulations at any treatment using one way ANOVA.

In VAD-AD and VAS-PF-4M ZL hepatocytes, insulin at 0.1 nM suppressed *Pck1* expression of by 60% and 70%, respectively (Figure 6A). The insulin-mediated suppression reached over 90% at 1 to 100 nM (left panel). In VAS-PF-AD ZL hepatocytes, the *Pck1* expression levels in the presence of 1 to 100 nM insulin were significantly higher than that in VAD-AD and VAS-PF-4M ZL hepatocytes (marked by * and $, respectively). In the presence of RA, insulin at 0.1 to 100 nM reduced the elevated *Pck1* mRNA levels in all three groups of ZL hepatocytes (Figure 6A, right panel).

For ZF hepatocytes, insulin at 0.1 to 100 nM significantly suppressed the *Pck1* expression in VAD-AD, VAS-PF-AD and VAS-PF-4M groups (Figure 6B, left panel). With RA, insulin reduced the elevated *Pck1* mRNA levels in VAS-PF-AD (0.1 to 100 nM), VAD-AD (1 to 100 nM) and VAS-PF-4M (1 to 100 nM) ZF hepatocytes (right panel). Interestingly, the *Pck1* expression levels in VAS-PF-AD ZF hepatocytes were significantly lower than those in VAD-AD ZF hepatocytes at every concentration (marked by *).

### The insulin-induced *Srebp-1c* expression was impaired by transient overeating in hepatocytes of VAS-PF-AD ZL and ZF rats

**Figure 7:**
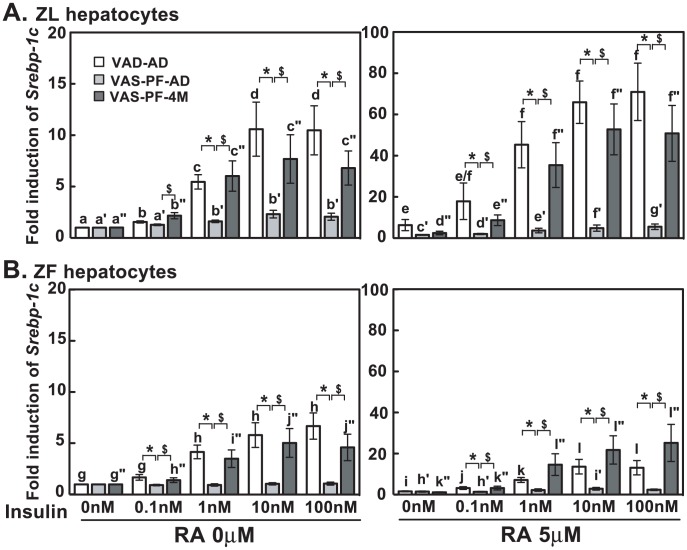
Transient overfeeding of VAS diet impaired insulin-induced *Srebp-1c* expression in hepatocytes of VAS-PF-AD rats. The primary hepatocytes were treated by insulin (0 nM to 100 nM) with or without RA (5 µM) in medium A for 6 hours. The expression level of *Srebp-1c* was determined by real-time PCR analysis, and the data were expressed as fold induction. The gene transcript levels from the no treatment group (0 nM insulin, no RA) for both ZL and ZF were arbitrarily set to 1. All p<0.05; for (A), a<b<c<d, a′<b′, a″<b″<c″, e<f, c′<g′/f′, d′<f′, d″<e″<f″; for (B), g<h, g″<i″/j″, h″<j″, i<k/l, j<l, h′<l′, k″<l″, using one way ANOVA. *, #, and $ for comparing the effects of dietary manipulations at any treatment using one way ANOVA.

In VAD-AD and VAS-PF-4M ZL hepatocytes, insulin at 0.1 to 100 nM induced the *Srebp-1c* expression with or without RA (Figure 7A). The fold inductions of *Srebp-1c* by insulin (1 to 100 nM) and insulin + RA (10 to 100 nM) in VAS-PF-AD ZL hepatocytes were significantly lower than that in VAD-AD (marked by *) and VAS-PF-4M (marked by $) ZL hepatocytes at the corresponding treatments.

In VAD-AD and VAS-PF-4M ZF hepatocytes, insulin at 1 to 100 nM induced the *Srebp-1c* expression with or without RA (Figure 7B). In contrast, the insulin-mediated inductions of *Srebp-1c* expression were abolished in VAS-PF-AD ZF hepatocytes regardless of RA. As a result, the fold inductions in VAS-PF-AD ZF hepatocytes were significantly lower than that in VAD-AD (marked by *) and VAS-PF-4M ZF (marked by $) hepatocytes. These data demonstrate that the transient overeating of VAS-PF-AD rats impairs the insulin-induced *Srebp-1c* expression in their hepatocytes.

### Dietary manipulation and VA status regulated the expression levels of genes for VA metabolism in hepatocytes from ZL and ZF rats

**Figure 8:**
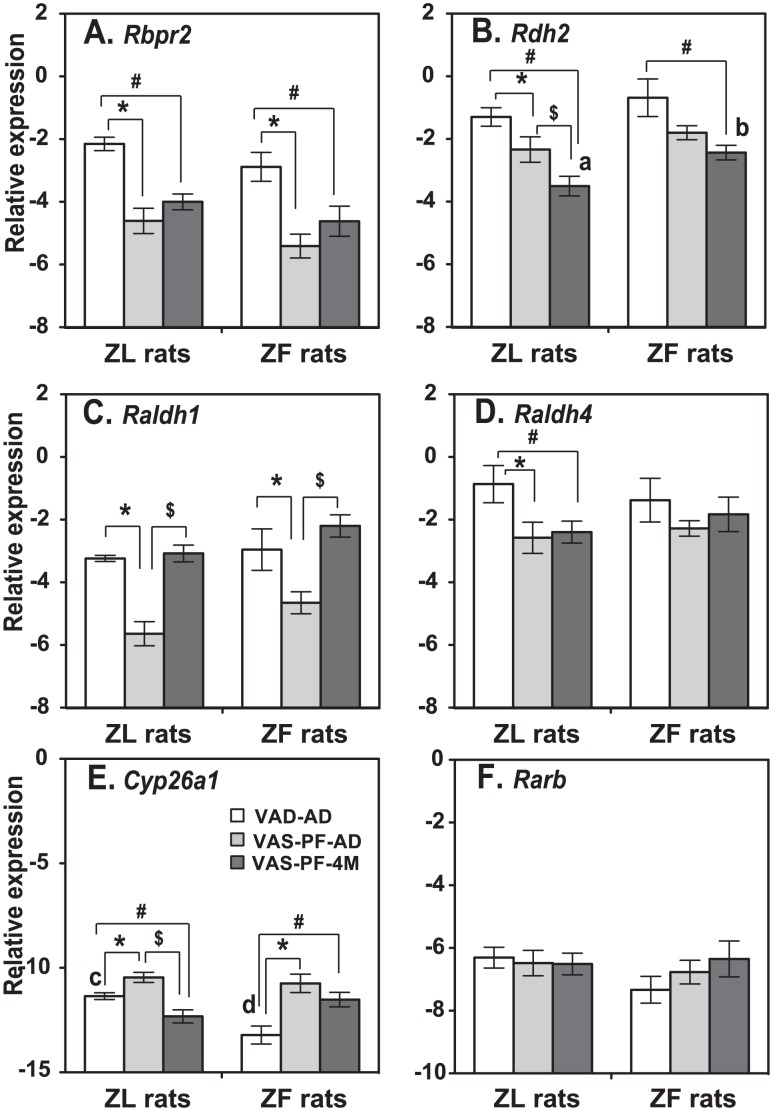
Differential expression levels of vitamin A metabolic genes in ZL and ZF primary hepatocytes. The expression levels of *Rbpr2* (A), *Rdh2* (B), *Raldh1* (C), *Raldh4* (D), *Cyp26a1* (E), and *Rarb* (F) were determined by real-time PCR in the cultured primary hepatocytes receiving no treatment. The data were expressed as −ΔCt (36B4 - gene of interest). All p<0.05; *, #, and $ for comparing the effects of dietary manipulation variables on gene transcript levels using two-way ANOVA with Bonferroni's post-hoc test; a>b, and c>d, using Student's t-test to compare ZL with ZF with the corresponding dietary manipulation.

To understand the impact of transient overeating on the hepatic VA metabolism, we analyzed the expression levels of key enzymes for VA metabolism and RA responses in all three groups of ZL and ZF hepatocytes. As shown in Figure 8A, the expression level of RBP4 receptor 2 (*Rbpr2*), a proposed liver-specific retinol transporter [17], was higher in VAD-AD hepatocytes than that in VAS-PF-AD and VAS-PF-4M hepatocytes (both ZL and ZF). RDH2 catalyzes the reversible conversion between retinol and retinal [18]. In Figure 8A, the expression level of *Rdh2* in VAD-AD ZL or ZF hepatocytes was higher than that in VAS-PF-4M ZL or ZF hepatocytes, respectively. The expression level of *Rdh2* in VAS-PF-AD ZL hepatocytes was higher than that in VAS-PF-4M, but lower than that in VAD-AD ZL hepatocytes.

Both RALDH1 (also known as ALDH1A1) and RALDH4 are involved in the irreversible conversion of retinal into RA [18]. The *Raldh1* (*Aldh1a1*) expression level in VAS-PF-AD ZL or ZF hepatocytes was significantly lower than that in VAD-AD and VAS-PF-4M ZL or ZF hepatocytes, respectively (Figure 8C). The *Raldh4* expression level in VAD-AD ZL hepatocytes was higher than that in VAS-PF-AD and VAS-PF-4M groups (Figure 8D), suggesting the alteration of VA metabolism in the liver. However, the *Raldh4* expression levels in all groups of ZF hepatocytes were similar.

Additionally, the expression level of *Cyp26a1* (Figure 8E), a RA catabolic gene [16], [19], in VAD-AD ZL hepatocytes was significantly lower than that in VAS-PF-AD, but higher than that in VAS-PF-4M ZL hepatocytes. The *Cyp26a1* expression level in VAD-AD ZF hepatocyte was lower than that in VAS-PF-AD and VAS-PF-4M ZF groups. The *Rarb* expression levels were not different among VAD-AD, VAS-PF-AD, and VAS-PF-4M groups (Figure 8F).

Two-way ANOVA analysis showed that dietary manipulations accounted for the majority of overall variance observed in the expression levels of *Rbpr2* (F = 23.04, 61% variance explained), *Rdh2* (F = 13.14, 47%), *Raldh1* (F = 23.27, 61%), and *Raldh4* (F = 3.56, 22%). This suggests that dietary manipulation can potentially change the availability of RA in primary hepatocytes by changing the expression levels of VA metabolic genes. Aside from the genes described above, we also checked *Stra6*, *Rdh10*, *Raldh2* and *Raldh3*, whose transcript levels in primary hepatocytes were too low to be determined by real-time PCR analysis.

## Discussion

Elucidating the molecular mechanisms resulting in hepatic insulin resistance is of great importance. It helps understand the development and progression of metabolic diseases. We investigated the effects of food intake and VA status on the insulin-regulated gene expression in primary hepatocytes of ZL and ZF rats. ZF rats, which bear a loss-of-function leptin receptor gene, develop obesity, dyslipidemia and insulin resistance due to hyperphagia [20]. We found that the insulin-regulated *Gck*, *Pck1* and *Srebp-1c* expressions were impaired in the primary hepatocytes of ZF rats fed chow diet ad libitum (Figure 1), confirming our previous observation [9]. VA deficiency partially corrected the impairment (Figure 2). We have reported that retinoids affect the expression levels of several insulin-regulated genes in primary hepatocytes previously. These observations are somewhat anticipated and in line with our hypothesis that both hyperinsulinemia and RA overproduction may take part in the regulation of the expression of genes for glucose and lipid metabolism, and contribute to hepatic insulin resistance at the gene expression level [1].

Compared to ZL rats, ZF rats are hyperinsulinemic [21]. Previous research showed that the insulin binding capacity of the isolated liver plasma membrane of ZF rats was not different from that of ZL rats [22]. In addition, the insulin-induced phosphorylation of AKT at Ser473 and Thr308 were similar in the primary hepatocytes of ZL and ZF rats [9]. These data indicate that the impairment of insulin-regulated gene expression in ZF hepatocytes is likely to be caused by alterations downstream of AKT activation in the insulin signaling cascade. Indeed, VA and insulin operate through separated pathways to regulate hepatic gene expression. RA, as a metabolite of VA catabolism, moves to the nucleus and activates the nuclear receptors (e.g. retinoic acid receptor, retinoid X receptor, peroxisome proliferator-activated receptor β/δ, hepatocyte nuclear factor 4α, and chicken ovalbumin up-stream transcription factor II) that are located in the promoters of the target genes [1]. How these two pathways interact with each other in normal or disease states deserves further investigation.

We also found that the degree of the impairment of *Srebp-1c* expression was more profound than that of *Gck* and *Pck1* in ZF hepatocytes (Figure 1). It suggests that branching points may exist in the downstream of insulin signaling pathway, below which the expressions of *Gck*, *Pck1* and *Srebp-1c* are differentially regulated by nutritional and hormonal stimuli. This is in accordance with other studies. For example, a bifurcation point at mTORC1 in the insulin signaling pathway separated the insulin-induced *Srebp-1c* expression and the insulin-suppressed *Pck1* expression [23]. The knockout of SREBP cleaving-activating protein gene in *ob/ob* mice resulted in the amelioration of hepatic lipogenesis without the improvement of hepatic gluconeogenesis [24]. Additionally in primary rat hepatocytes treated with insulin, the *Gck* expression reached the peak level 6 hours earlier than did the *Srebp-1c* expression [10]. Further studies are needed to dissect these branching points and discover any change of regulation in insulin resistance.

The negative effects of short-term overfeeding on the hepatic insulin sensitivity have been observed in both rodents and humans with unrevealed mechanisms. The pair-feeding study indicates that the transient overeating of the VAS diet caused the impairment of insulin-regulated *Gck*, *Pck1* and *Srebp-1c* expressions in the primary hepatocytes of ZL rats (Figure 5, 6, 7). In contrast, VAS-PF-4M ZL rats showed normal insulin-regulated gene expression. This suggests that, in the liver of so called insulin-sensitive ZL rats, insulin sensitivity at gene expression level can be acutely impaired after excessive food intake, and then restored overtime. Indeed, in young human subjects, a 7-day overfeeding increased circulating adiponectin and glucagon-like peptide 1 levels, which are proposed to act against the hepatic insulin resistance [25], [26]. On the other hand, transient overeating of the VAS diet caused worsened impairment of insulin-regulated *Gck* and *Srebp-1c* expressions in the primary hepatocytes of ZF rats (Figure 5, 7). ZF rats persist in overeating state due to deficient leptin signaling. This may hamper the restoration of insulin sensitivity after food intake. As a result, ZF rats fed VAS or chow ad libitum manifest the hepatic insulin resistance at the gene expression level.

Long term VA deficiency decreased the insulin-regulated expression of *Gck*, but not that of *Pck1* and *Srebp-1c*. In VAS-PF-AD ZL rats, the insulin-induced *Gck* expression was significantly lower than that in VAS-PF-4M ZL rats (Figure 5), showing that adequate VA status is instrumental to the insulin-regulated *Gck* expression in insulin-sensitive ZL primary hepatocytes. This observation demonstrates that VA status differentially affects the hepatic insulin-regulated gene expression, suggesting complex interactions of VA and insulin signaling systems.

The impairment of insulin-induced *Gck* (Figure 5) and insulin-suppressed *Pck1* expressions (Figure 6) in VAS-PF-AD hepatocytes suggests an elevation of plasma glucose level and an acute loss of insulin-sensitivity in over-feeding state. This is supported by the elevated tail tip whole blood glucose level observed in these animals (Figure 3E), and may explain the elevated hepatic glucose production in human subjects who are overfed for short terms [27], [28]. The dysregulation of insulin-induced hepatic *Srebp-1c* expression after the transient overeating (Figure 7) suggests increased *de novo* lipogenesis, which may explain the increased hepatic fat content observed in short-term overfed humans [29]. More importantly, the impairment was observed in both ZL and ZF rats after transient overeating. These data suggest the existence of a mechanism that may dynamically regulate hepatic insulin sensitivity at the gene expression level in response to feeding status.

Short-term overeating provides excessive intake of not only calories but also micronutrients. We showed transient overeating for one day greatly affected the expression levels of genes involved in VA metabolism in primary hepatocytes of ZL and ZF rats (Figure 8). VAD-AD rats exhibited high hepatic levels of *Rbpr2*, *Rdh2*, *Raldh1 (Aldh1a1)* and *Raldh4* transcripts, showing compensatory upregulation of the VA metabolic genes. In contrast, VAS-PF-4M rats exhibited low hepatic transcript levels of *Rbpr2*, *Rdh2* and *Raldh4*. These suggest that the hepatic VA metabolic gene expression is greatly influenced by the availability of dietary VA.

VAS-PF-AD rats had lowered hepatic levels of *Rbpr2* and *Raldh1 (Aldh1a1)* transcripts. Indeed, the *Rbpr2* expression is inversely related to the liver retinol stores, and RA reduces the *Rbpr2* mRNA level in HepG2 cells [17]. Additionally, the elevated RA controls its biosynthesis by down-regulating RALDH1 (ALDH1A1) through the modulation of retinoic acid receptor α and CCAAT/enhancer binding protein β [30], [31]. These suggest that the expression levels of VA metabolic genes are modulated in VAS-PF-AD rats to prevent the RA overproduction in response to the transient influx of the dietary VA. Despite the negative feedback regulation, excessive RA may have been produced. This is supported by the increased expression level of *Cyp26a1* [19], a RA-responsive gene, in VAS-PF-AD hepatocytes (Figure 8E). The excessive RA may affect the expression of hepatic metabolic genes, and promote the hepatic insulin resistance at the gene expression level [1]. It is interesting to note that the VA status does not seem to affect the expression levels of *Raldh1* (*Aldh1a1*) gene in primary hepatocytes as its expression levels are similar in VAD-AD and VAS-PF-4M groups. The underlying mechanism deserves further investigation.

In summary, we have demonstrated that both VA and feeding statuses affected the hepatic insulin sensitivity at the gene expression level. This finding provides insight into the development of the hepatic insulin resistance, and helps find solutions to combat metabolic diseases.

## Supporting Information

File S1 (PDF)
