# Supplementary material for: Vitamin A and Feeding Statuses Modulate the Insulin-Regulated Gene Expression in Zucker Lean and Fatty Primary Rat Hepatocytes
Source: PLoS One. 2014 Aug 8;9(8):e100868. doi: 10.1371/journal.pone.0100868 (PMC4126667; doi:10.1371/journal.pone.0100868)
Supplement: File S1 — (PDF) [file pone.0100868.s001.pdf]

SUPPORTING ONLINE DATA

Vitamin A and feeding statuses modulate the insulin-regulated gene expression in Zucker lean and fatty primary rat hepatocytes

Wei Chen, Meredith L. Howell, Yang Li, Rui Li and Guoxun Chen

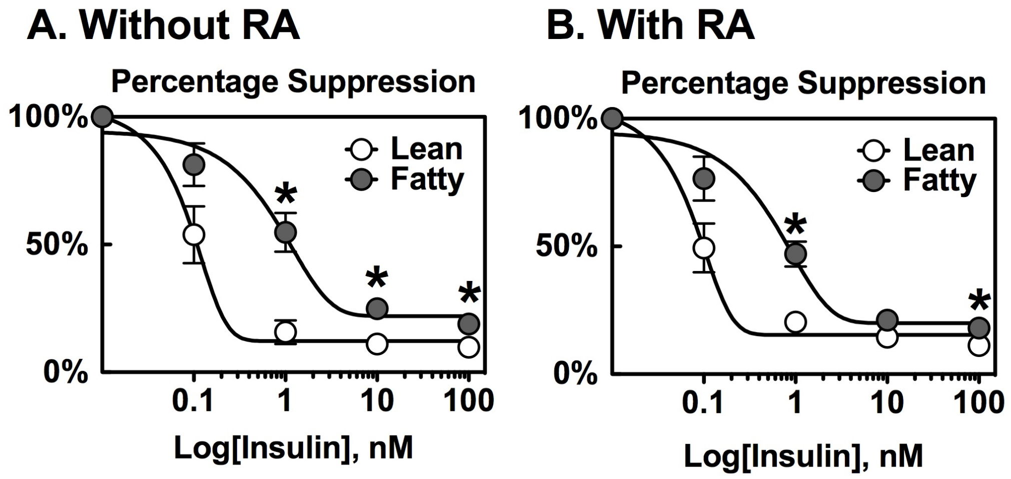

Supporting Figure S1: The impaired insulin-suppressed *Pck1* expression in primary hepatocytes isolated from ZF, but not ZL, rats fed chow ad libitum. ZL and ZF rats were fed standard chow for eight weeks before primary hepatocytes were harvested. The primary hepatocytes were incubated in medium A with increasing concentrations of insulin (0nM to 100nM) in the absence or presence of RA (5µM) for 6 hours. Total RNA was extracted, synthesized into cDNA, and then subjected to real-time PCR analysis. The expression level of *Pck1* in ZL or ZF hepatocytes treated with vehicle control was arbitrarily set to 100%. The data were expressed as percentage suppression. All *$p<0.05$ for comparing ZL or ZF at corresponding treatments using Student's t-test.

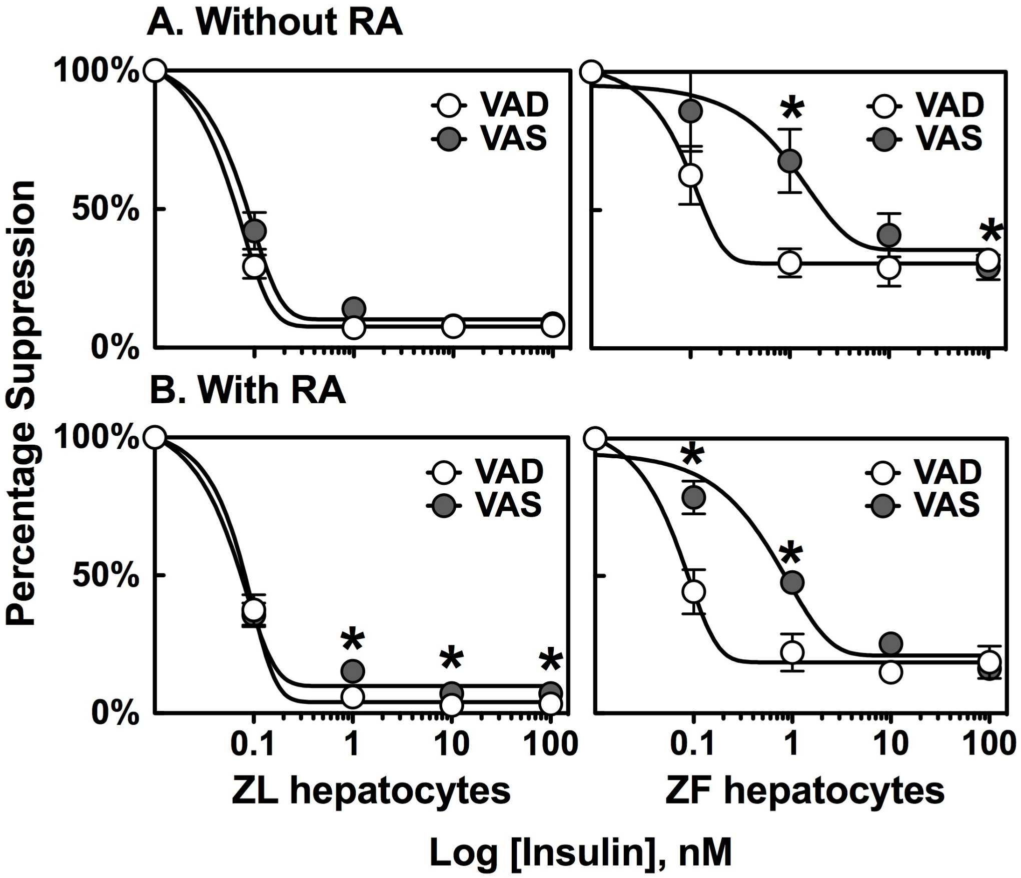

Supporting Figure S2: The impaired insulin-suppressed *Pck1* expression in primary hepatocytes from ZF rats was partially recovered in rats fed a VAD diet for 8 weeks. ZL and ZF rats were fed either a VAS or VAD diet for 8 weeks. Primary hepatocytes were isolated from rats in ad libitum. Cells were incubated in medium A with increasing concentrations of insulin (0nM to 100nM) in the absence or presence of RA (5µM) for 6 hours. Total RNA was extracted, synthesized into cDNA, and then subjected to real-time PCR analysis. The expression level of *Pck1* in ZL or ZF hepatocytes treated with vehicle control was arbitrarily set to 100%. The data were expressed as percentage suppression. All *$p<0.05$ for comparing ZL or ZF at corresponding treatments using Student's t-test.
